# Integration of Behavioral Health by Community Health Workers Into a Lifestyle Intervention That Addresses Reduction of Risk for Cardiovascular Disease in Rural Communities

**DOI:** 10.5888/pcd23.260142

**Published:** 2026-10-08

**Authors:** Stephanie Coronel-Mockler, Nick Flattery, Ashley Ambrose, Samuel Hubley, Kristin Kilbourn, Carter Sevick, Emily Bilenduke, Bryan Contreras Zamora, Jacqueline Howard, Raymond O. Estacio

**Affiliations:** 1 Colorado Prevention Center Community Health, Aurora, Colorado; 2 Renée Crown Wellness Institute, University of Colorado Boulder; 3 Department of Psychology and Neuroscience, University of Colorado Boulder; 4 Department of Psychology, University of Colorado Denver; 5 Department of Medical Social Sciences, Northwestern University Feinberg School of Medicine, Chicago, Illinois; 6 Division of General Internal Medicine, School of Medicine, University of Colorado Anschutz, Aurora, Colorado

## Abstract

**Purpose and Objectives:**

Cardiovascular disease (CVD) is the leading cause of death and disability in the US, with higher rates in rural areas. Although community health workers (CHWs) are effective in improving CVD outcomes, limited evidence exists on their role in addressing behavioral health needs within lifestyle change interventions. This evaluation assessed the feasibility of integrating behavioral health components focusing on psychologic distress, anxiety, and depression into the Colorado Heart Healthy Solutions program (CHHS) in rural communities by using 3 domains of the RE-AIM (Reach, Effectiveness, Adoption, Implementation, and Maintenance) framework.

**Intervention Approach:**

Beginning in 2018, behavioral health components were added to CHHS: assessments for stress, anxiety, and depression; connections with community-based behavioral health resources; and a health coaching intervention linking physical and mental health. Program adaptations that provided additional CHW training, lowered screening thresholds, and expanded intervention materials were implemented to address local determinants.

**Evaluation Methods:**

Reach, implementation, and maintenance were assessed by using completion rates of each behavioral health component from 2019 to 2023. Logistic regression within an interrupted time-series framework evaluated the effects of program adaptations on CHW delivery.

**Results:**

CHWs completed 8,077 visits with 5,559 participants in 7 rural communities. Completion rates during the first 10 months ranged from 78.6% to 87.5% for the behavioral health assessments; 51.9% completed the connections to care and 8.2% completed the coaching intervention. Program adaptations in 2019 significantly improved completion of the brief assessments and coaching intervention use (*P* < .001). Adaptations in 2022 increased intervention use (*P* < .001) but initially reduced completion of longer assessments. By the final year, assessment completion rates were above 89%, with intervention delivery reaching 42.8%.

**Implications for Public Health:**

Integrating behavioral health into a CHW-led program in rural communities was feasible and sustainable. High completion rates suggest that CHWs can effectively deliver behavioral health assessments and support in underserved settings, highlighting the value of flexible, community-informed implementation strategies.

**Summary**

**What is already known on this topic?**

Community health workers (CHWs) are effective in improving cardiovascular disease risk factors and health behaviors. However, data on CHWs addressing behavioral health within a lifestyle change program in rural areas are limited.

**What is added by this report?**

This evaluation used 3 dimensions of the RE-AIM (Reach, Effectiveness, Adoption, Implementation, and Maintenance) framework to demonstrate the feasibility of CHWs delivering behavioral health components within a lifestyle change program in rural communities.

**What are the implications for public health practice?**

This evaluation provides key insights to optimize implementation and emphasizes the importance of a flexible, community-informed approach with ongoing adjustments to improve and sustain fidelity.

## Purpose and Objectives

Cardiovascular disease (CVD) continues to be the leading cause of death and disability in the US, with higher rates in rural areas compared with urban areas (1). 2017 Data from the Centers for Disease Control and Prevention showed heart disease prevalence of 14.2% among rural residents versus 11.2% in small metropolitan areas and 9.9% in urban areas (2). From 2010 to 2022, CVD mortality rates declined in urban areas but increased in rural areas, particularly among younger adults (3).

High CVD mortality rates in rural areas reflect the combined influence of biological, psychological, and social factors. Biologically, rural populations have higher rates of hypertension, obesity, diabetes, tobacco use, and poor diet, all of which contribute to increased CVD risk (4,5). Psychologic risk factors — including stress, depression, and anxiety — intensify CVD risk by undermining health behaviors, decreasing medication adherence, and activating maladaptive physiologic stress pathways (6–8). Additionally, people in rural communities have a substantially increased risk of depression, substance use disorders, and suicide (9). Rural residents tend to have lower rates of mental health care use than urban residents, as well as significantly higher rates of suicide and deaths associated with substance use (10).

Worsening these conditions in rural areas are social factors that include high poverty rates, limited access to health care, long travel distances to access care, a shortage of health care providers, and less social support (5). In addition, social factors such as stigma surrounding mental illness, low levels of mental health literacy, and social isolation exacerbate mental health challenges (11). These factors underscore the need for comprehensive programs in rural communities to narrow the rural–urban gap in both mental health and CVD risk and mortality. A historical change to health care delivery as a result of the COVID-19 pandemic has led to exacerbated urban–rural disparities in CVD mortality rates (3). The factors associated with this widening gap include strained health care systems, telehealth inequities, and higher rates of psychosocial comorbidities (12).

Community health workers (CHWs) have become essential in closing the gaps in access to health care (13–15). As trained public health professionals with lived experience in the communities they serve, they deliver culturally tailored outreach and education, offer informal counseling and social support, and advocate for their communities. By integrating local values into every interaction, CHWs effectively bridge the divide between underserved populations and the health or social services needed (16,17).

In 2009 the Colorado Heart Healthy Solutions (CHHS) program was developed to reduce the prevalence of CVD by using CHWs to deliver a community-based, lifestyle change program at no cost to participants (14). The CHHS program is a partnership between the Community Health team of the Colorado Prevention Center (CPC), which is a nonprofit, academically led organization, and local public health agencies, clinics, and community health organizations in rural counties. Counties were categorized as rural based on the Colorado Rural Health Center’s definition of a nonmetropolitan county containing no municipalities of more than 50,000 residents (18). Each partner organization used 1 or 2 CHWs to deliver the CHHS program in their community. With program-specific training and ongoing support from CPC, the CHWs implemented the intervention by conducting health screenings, providing education, encouraging positive behavior change through health coaching, and linking participants to local resources. Adult participants were recruited primarily via community outreach. Examples of outreach venues included local businesses, churches, community centers, and large-scale health fairs. Screenings for CVD risk included measurements for blood pressure, body mass index, point-of-care glucose and lipid levels (Abbott Laboratories), and an estimate of the person’s risk of a cardiovascular event over the next 10 years (19,20). CHWs also collected data on health behaviors, including diet, exercise, and smoking. From 2009 to 2018, the CHHS program demonstrated improvements in low-density lipoprotein cholesterol, systolic blood pressure, weight, and Framingham risk scores among participants at risk for CVD (14).

The Community Preventive Services Task Force recommends interventions that engage CHWs to prevent CVD (21). However, many CHW-led CVD prevention programs, including CHHS, historically did not address mental health risk factors such as anxiety, depression, and stress — despite their well-established influence on health behaviors and CVD outcomes. In 2018, we expanded CHHS to include behavioral health components after both quantitative and qualitative data from participating communities showed a substantial unmet need for mental health support that directly affects CVD risk and behavior change. In a local survey, 68% of CHHS participants reported being “very interested” in expanded behavioral health services to help manage participant-reported stress, anxiety, and depression. This participant feedback, combined with documented behavioral health workforce shortages in rural Colorado (22), prompted the integration of 3 components into CHHS: routine screening, referral pathways, and brief CHW‑delivered interventions. Although preliminary studies have demonstrated that CHWs can deliver mental health interventions that address disorders like depression and anxiety (13,23,24), data on CHWs addressing mental health topics as part of an overall lifestyle change program in rural areas are limited.

The goal of integrating behavioral health components that address stress, depression, and anxiety into the CHHS program was to equip CHWs with tools and skills to address both the physical and mental health of participants and ultimately improve CVD risk in rural communities. This evaluation assessed CHW delivery of the behavioral health components by detailing the components, adaptations made to respond to local needs, and subsequent effects of those adaptations on implementation outcomes.

The RE-AIM (Reach, Effectiveness, Adoption, Implementation and Maintenance) framework (25) is a well-established model for planning and evaluating health interventions. Designed to facilitate translation of research to practice, its comprehensive approach looks beyond effectiveness and incorporates the factors that influence uptake, delivery, and sustainability of interventions in real-world settings. Three RE-AIM dimensions guided the evaluation: reach, implementation, and maintenance. Reach assessed the percentage of CHHS participants who engaged with the behavioral health components, as well as the sociodemographic characteristics of people who participated and those who did not. Implementation assessed the fidelity of CHW delivery and component adaptations. Maintenance evaluated the extent to which delivery of the behavioral health components was sustained. CHHS was deemed nonhuman subjects research by the Colorado Multiple Institutional Review Board (no. 10-0368).

## Intervention Approach

### Behavioral health components

CHHS integrated behavioral health elements by using an established primary care framework that organizes integrated care into 8 domains (26). Guided by this framework, behavioral health components were added in 3 stages of the CHHS program: 1) screening assessments, 2) connections to care, and 3) brief CHW-delivered health coaching interventions.

Screening assessments for depression, anxiety, and stress included the Patient Health Questionnaire-2 (PHQ-2), Generalized Anxiety Disorder-2 (GAD-2) questionnaire, and the Perceived Stress Scale (PSS-4), respectively (27–29). These widely used screeners were selected for their brevity to reduce burden on both the participants and CHWs in community settings and for their validation in English and Spanish. Assessments for depression and anxiety use a 4-point Likert scale to measure severity of symptoms during the past 2 weeks (0 being “not at all” to 3 being “nearly every day”). The PSS-4 uses a 5-point Likert scale to measure how often people experience stressful situations in the past month (0 being “never” to 4 being “very often”). Participants who scored 3 or higher on the PHQ-2 or GAD-2 continued with the PHQ-8 (30) and GAD-7 (31) to assess additional symptoms of depression and anxiety.

To facilitate connections to care, participants were referred to local behavioral health provider, primary care, or telehealth counseling when they scored 10 or higher on the PHQ-8 or GAD-7, or at the discretion of the CHWs conducting the screening. An inventory of behavioral health resources in each community was identified before program implementation.

The intervention was designed to link physical and mental health for participants with elevated risk for CVD and an interest in exploring behavior change. Initially, “behavior maps” were incorporated into the motivational interviewing–based health coaching intervention. These maps were adapted from the behavioral activation component of cognitive behavioral therapy and were designed to visually depict patterns that link thoughts, feelings, and contextual triggers to unhealthy behaviors. Each map included 3 boxes: one for the unhealthy behavior the participant wanted to address, one for contextual triggers (events), and one for experiential triggers (thoughts and feelings).

During each visit, the CHW invited the participant to describe the behavior they wished to explore and helped organize the story into the 3 components of the map. Together, they identified how triggers contributed to short‑ and long‑term cycles that reinforced the behavior. The CHW then guided the participants in identifying a potential change that could interrupt the cycles.

### CHW training

CHWs received approximately 20 hours of initial training via a 3-day workshop led in 2018 by the multidisciplinary program team with expertise in public health, medicine, and clinical psychology (Figure 1). Training covered the relationship between mental health and CVD, how to explain and administer behavioral health assessments, how to initiate referrals, how to use the behavior maps, and data collection. Ongoing support included another 20 hours through monthly meetings and behavioral health–focused sessions with the clinical psychologist during 1 year.

**Figure 1:**
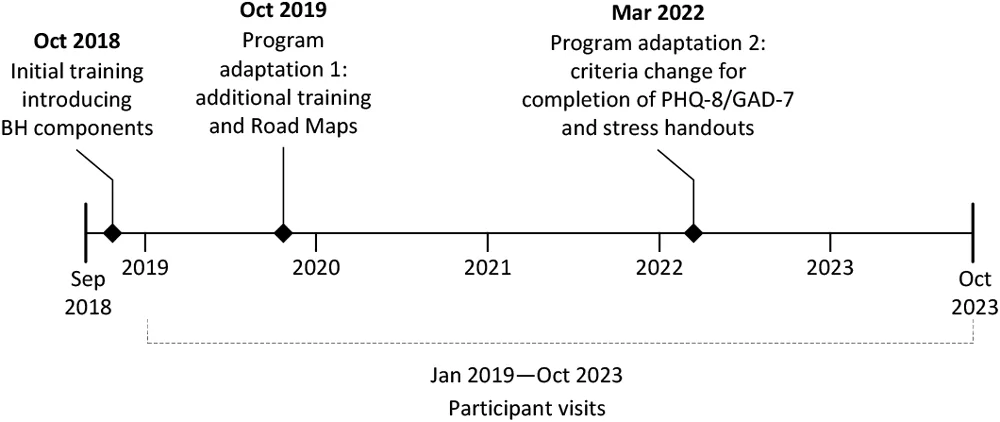
Timeline of program implementation of behavioral health components into a lifestyle intervention addressing reduction of risk for cardiovascular disease in rural communities, Colorado Heart Healthy Solutions, 2018–2023. Abbreviations: BH, behavioral health; GAD, Generalized Anxiety Disorder questionnaire; PHQ, Patient Health Questionnaire.

### Program adaptations

During the implementation period, several program adaptations were made in response to challenges observed by the program team, low completion rates identified during monthly data monitoring, and feedback shared by the CHWs during monthly team meetings. In response to declining behavioral health assessment completion rates, CHWs were given the flexibility to choose between self-administered paper forms or verbally administered questions to help address barriers such as time constraints, literacy levels, and privacy concerns. In response to the low delivery rates of the behavior map component, which were caused by time constraints reported by the CHWs, 7 “Road Maps for Change” were developed based on common behaviors and triggers. Each road map for change included a narrative that depicted a person’s struggle with a common unhealthy behavior, a corresponding behavior map of the narrative, and ideas for next steps based on readiness to change. In response to observations that participants were more comfortable talking about stress, stress management handouts were also developed to facilitate discussions about the connection between stress and unhealthy behaviors. These materials focused on the physical signs and long-term risks of stress, how to identify and adequately manage controllable and uncontrollable stressors, and general coping strategies. Finally, the PHQ-2 and GAD-2 thresholds for the full assessments were lowered from 3 or more to 2 or more due to lower-than-expected positive screen rates. These program adaptations occurred in 2 stages (Figure 1):

- October 2019: Training on barriers and best practices and the addition of “Road Maps for Change” handouts to the intervention (referred to as training/road maps).
- March 2022: Lowered PHQ-2/GAD-2 thresholds (score of ≥2) for full assessments and added psychoeducational stress management materials to the intervention (referred to as criteria change/stress handouts).

To support the work of the CHWs, CHHS uses the Outreach, Screening, and Referral (OSCAR) system, a data collection and decision-support tool that captures participant health data, tailors real-time evidence-based health messages using that data, and provides reminders when participants are due for follow-up visits (14). To support CHWs in delivering the behavioral health components, the OSCAR system was expanded to capture behavioral health assessment responses, calculate scores, prompt CHWs to administer the PHQ-8 and GAD-7, generate messages, recommend connections to care, and capture data on intervention use.

## Evaluation Methods

### Data collection

The data collection tool used for this quantitative evaluation was the OSCAR system. During participant visits, CHWs captured data on CVD risk factors and health behaviors, health history, responses to assessments, connections to care, and interventions used. Behavioral health assessment scores and CVD risk scores were automatically calculated and captured by the OSCAR system. Sociodemographic data were collected during the first visit.

### Measures

To assess the feasibility of CHWs delivering 3 behavioral health components within CHHS, RE-AIM domains focused on reach, implementation, and maintenance. Reach was conceptualized as the proportion of CHHS participants who engaged with 1 or more behavioral health components. A comparison was conducted of the sociodemographic characteristics of participants who did and did not complete any of the behavioral health components. Sociodemographic data included age, sex, race and ethnicity, health insurance, education, and employment status. Reach also measured the proportion of CHHS participants among the intended populations who engaged with individual behavioral health components. Intended populations were defined as the following: 1) all CHHS participants were eligible for the PHQ-2, GAD-2, and PSS-4 assessments; 2) CHHS participants who screened positive for depression on the PHQ-2 and were eligible for the PHQ-8 and those who screened positive for anxiety on the GAD-2 and were eligible for the GAD-7; 3) participants with elevated PHQ-8 scores, GAD-7 scores, or both (score of ≥10) were eligible for connections with care; and 4) participants with elevated CVD risk and who had an interest in exploring behavior change were prioritized for the behavioral health coaching interventions.

The implementation dimension measured completion rates of each behavioral health component at each visit to assess the delivery of each behavioral health component. Assessments were defined as complete when all items were rated. Connection to care was defined as complete when participants with elevated PHQ-8 or GAD-7 scores were referred to or had a connection to a behavioral health resource. Intervention completion was defined as the completion of a behavior map, a Road Map for Change, or a stress management intervention.

Maintenance evaluated the extent to which behavioral components were sustained during each year. Benchmarks were defined as greater than 85% completion of behavioral health assessments, 50% or more of participants with elevated PHQ‑8 or GAD‑7 scores being connected to care, and the coaching intervention delivered in 40% or more of visits with participants at elevated CVD risk. These thresholds were set based on sustainment concepts from implementation frameworks (32), CHW input based on capacity to ensure targets were achievable in rural community settings, and implementation science standards emphasizing pragmatic, feasible sustainment thresholds in real-world settings (33).

### Data analysis

We summarized sociodemographic characteristics of the CHHS participants by using frequency distributions for categorical variables. We compared sociodemographic variables between the group who engaged in the behavioral health components and those who did not by using the χ^2^ test for categorical variables. Completion rates for assessments, connections, and intervention were summarized by year. The impact of the 2 program adaptations was evaluated by using logistic regression within an interrupted time-series (ITS) framework (34). Multiple participant visits were accounted for by using generalized estimating equations (35). Initial models were built assuming the effects of the interruptions were immediate. However, when visual inspection of fit plots indicated that a period of adoption and adjustment may have occurred, additional data-driven analyses were performed to estimate this period. To facilitate this, models were estimated with an additional interruption parameter in 2-week intervals after the initial event. The interruption point producing the greatest benefit to the quasi-likelihood under the independence model information criterion was selected into the model (36). Effect estimates were presented as odds ratios (ORs) with 95% CIs and *P* values to convey precision. Data management and analyses were performed by using SAS version 9.4 (IBM Corporation).

## Results

### Reach

From 2019 to 2023, CHWs completed 8,077 visits with 5,559 unique CHHS participants across 7 rural communities. These participants included a substantial representation of Hispanic residents (34.0%), people without health care coverage (15.5%), and people without a medical home (20.9%) (Table 1). Among the 4,958 (89.2%) participants who engaged in 1 or more behavioral health components, 91.2% of non-Hispanic White participants completed the behavioral health component compared with 85.7% of Hispanic participants. Similarly, 89.8% of those whose preferred language was English completed the behavioral health component compared with 84.7% of those who preferred Spanish. Female participants (90.8%) and those with higher educational levels (89.4%) were also significantly more likely to complete the behavioral health component than male participants (86.6%) and those with lower educational levels (78.2%).

**Table 1:** Sociodemographic Characteristics of Participants in a Lifestyle Intervention to Reduce Risk of CVD in Rural Communities, January 2019–October 2023, Colorado Heart Healthy Solutions

Characteristic	All CHHS participants, no. (%) (N = 5,559)^a^	Participants who completed ≥1 behavioral health component, no. (%) (n = 4,958)	*P* value
**Age, y**			
18–39	1,375 (24.7)	1,221 (88.8)	.50
40–64	2,723 (49.0)	2,442 (89.7)	
≥65	1,460 (26.3)	1,294 (88.6)	
**Sex**			
Female	3,410 (61.3)	3,098 (90.8)	<.001
Male	2,148 (38.6)	1,860 (86.6)	
**Race and ethnicity**			
Hispanic	1,892 (34.0)	1,622 (85.7)	<.001
Non-Hispanic White	3,366 (60.6)	3,071 (91.2)	
Other	221 (4.0)	196 (88.7)	
**Education**			
Less than high school diploma	353 (6.4)	276 (78.2)	<.001
High school diploma or higher	3,535 (63.6)	3,159 (89.4)	
**Employment status**			
Employed	3,529 (63.5)	3,161 (89.6)	.60
Unemployed or unable to work	508 (9.1)	448 (88.2)	
Restricted income	1,178 (21.2)	1,049 (89.0)	
**Preferred language**			
English	4,960 (89.2)	4,453 (89.8)	<.001
Spanish	587 (10.6)	497 (84.7)	
**Health care coverage**			
No	862 (15.5)	734 (85.2)	<.001
Yes	4,634 (83.4)	4,181 (90.2)	
**Medical home**			
No	1,163 (20.9)	969 (83.3)	<.001
Yes	4,390 (79.0)	3,983 (90.7)	
**At risk for CVD**			
No	292 (5.3)	256 (87.7)	.29
Yes	4,908 (88.3)	4,398 (89.6)	

Abbreviations: CHHS, Colorado Heart Healthy Solutions; CVD, cardiovascular disease.

a Data were collected by community health workers during an initial visit. Not all participants answered all questions; numbers may not add to 5,559 and percentages to 100 because of missing responses.

Engagement with individual behavioral health components was also high. Of the 5,559 participants served, 4,871 (87.6%) completed at least 1 PSS-4 assessment; 4,888 (87.9%) completed a PHQ-2 assessment, and 4,888 (87.9%) completed a GAD-2 assessment. Of the 446 participants who screened positive for depression on the PHQ-2, 418 (93.7%) completed a PHQ-8. Of the 727 participants who screened positive for anxiety on the GAD-2, 651 (89.5%) completed the GAD-7. Of the 406 participants who had scores of 10 or higher on the PHQ-8, the GAD-7, or both, 215 (53.0%) were connected with care. Of the 2,433 participants with elevated CVD risk and an interest in behavior change, 1,036 (42.6%) received the intervention.

### Implementation

Completion rates for the PSS-4, PHQ-2, and GAD-2 assessments during the initial implementation period between January and October 2019 ranged from 78.6% to 79.9% (Table 2). Results from the ITS analysis showed a decline in completion rates during this time frame (Figure 2A–C). However, all 3 assessments had increased uptake (*P* < .001) after the program adaptations made in year 1 (additional training/road maps), as demonstrated by the 5-fold increase in the odds of completion compared with the initial implementation period (Table 2 and Figure 2A–C). Lowering the criteria for the PHQ-8 and GAD-7 assessments as part of program adaptations in year 4 (criteria change/stress handouts) initially led to a decline in the completion rates for the PHQ-8 (OR = 0.09; 95% CI, 0.01–0.77; *P* = .03) and GAD-7 (OR = 0.31; 95% CI, 0.09–1.09; *P* = .07). However, completion rates for both the PHQ-8 and GAD-7 continued to improve (Figure 2D–E) and returned to 93.5% and 89.2%, respectively, after a second adaptation while increasing the number of participants being fully assessed for symptoms of depression and anxiety (Table 2).

**Table 2:** Completion Rates of Behavioral Health Components Pre- and Post-Program Adaptations and Change in Odds of Completion Post-Program Adaptations, Colorado Heart Healthy Solutions

Behavioral health component	Completion rates, 2019–2023				Change in odds of completion	
	Initial implementation (January–October 2019)	PA no. 1: training/intervention expansion (October 2019–March 2022)^a^	PA no. 2: criteria change/intervention expansion (March 2022–October 2023)^b^	All years	PA improving odds of completion	Change in odds of completion, OR (95% CI) [*P* value]
Total no.	1,431	3,674	2,972	8,077	—	—
**Perceived Stress Score 4-item assessment**						
No. (%)	1,124 (78.6)	3,157 (85.9)	2,653 (89.3)	6,934 (85.8)	PA 1	5.48 (4.04–7.45) [<.001]
**PHQ 2-item assessment**						
No. (%)	1,140 (79.7)	3,176 (86.5)	2,650 (89.2)	6,966 (86.2)	PA 1	5.40 (3.97–7.34) [<.001]
**GAD 2-item assessment**						
No. (%)	1,143 (79.9)	3,176 (86.5)	2,652 (89.2)	6,971 (86.3)	PA 1	5.36 (3.94–7.30) [<.001]
**PHQ-8**						
Eligible, no.^c^	40	127	325	492	PA 2	0.09 (0.01–0.77) [.03]
No. (%)	35 (87.5)	121 (95.3)	304 (93.5)	460 (93.5)		
**GAD-7**						
Eligible, no.^d^	96	209	518	823	PA 2	0.31 (0.09–1.09) [.07]
No. (%)	82 (85.4)	195 (93.3)	462 (89.2)	739 (89.8)		
**Connection to care**						
Eligible, no.^e^	79	197	181	457	NA	
No. (%)	41 (51.9)	106 (53.8)	90 (49.7)	237 (51.9)		
**Coaching intervention**						
Eligible, no.^f^	401	1,240	1,589	3,230	PA 1	8.99 (4.51–17.93) [<.001]
No. (%)	33 (8.2)	504 (40.6)	680 (42.8)	1,217 (37.7)	PA 2	1.94 (1.42–2.63) [<.001]^g^

Abbreviations: —, not applicable; GAD, Generalized Anxiety Disorder questionnaire; ITS, interrupted time series; NA, no indication found to pursue ITS; OR, odds ratio; PA, program adaptation; PHQ, Patient Health Questionnaire.

a PA 1 consisted of additional training and expansion of intervention with “Road Maps for Change” handouts.

b PA 2 consisted of 1) a criteria change to complete the PHQ-8 and/or the GAD-7 from a score of 3 or more on the PHQ-2 or the GAD-2 to a score of 2 or more and 2) expansion of the intervention with stress management handouts.

c Participants whose PHQ-2 scores met official criteria for depression.

d Participants whose GAD-2 scores met official criteria for anxiety.

e Participants with PHQ-8 or GAD-7 scores ≥10.

f Participants who were both at elevated risk for cardiovascular disease and who had an interest in behavior change.

g Comparison of the odds of completion between PA 1 and PA 2 after PA 2 was added to the coaching intervention. However, any effect that PA 1 might have had was hypothetical and not tested.

**Figure 2:**
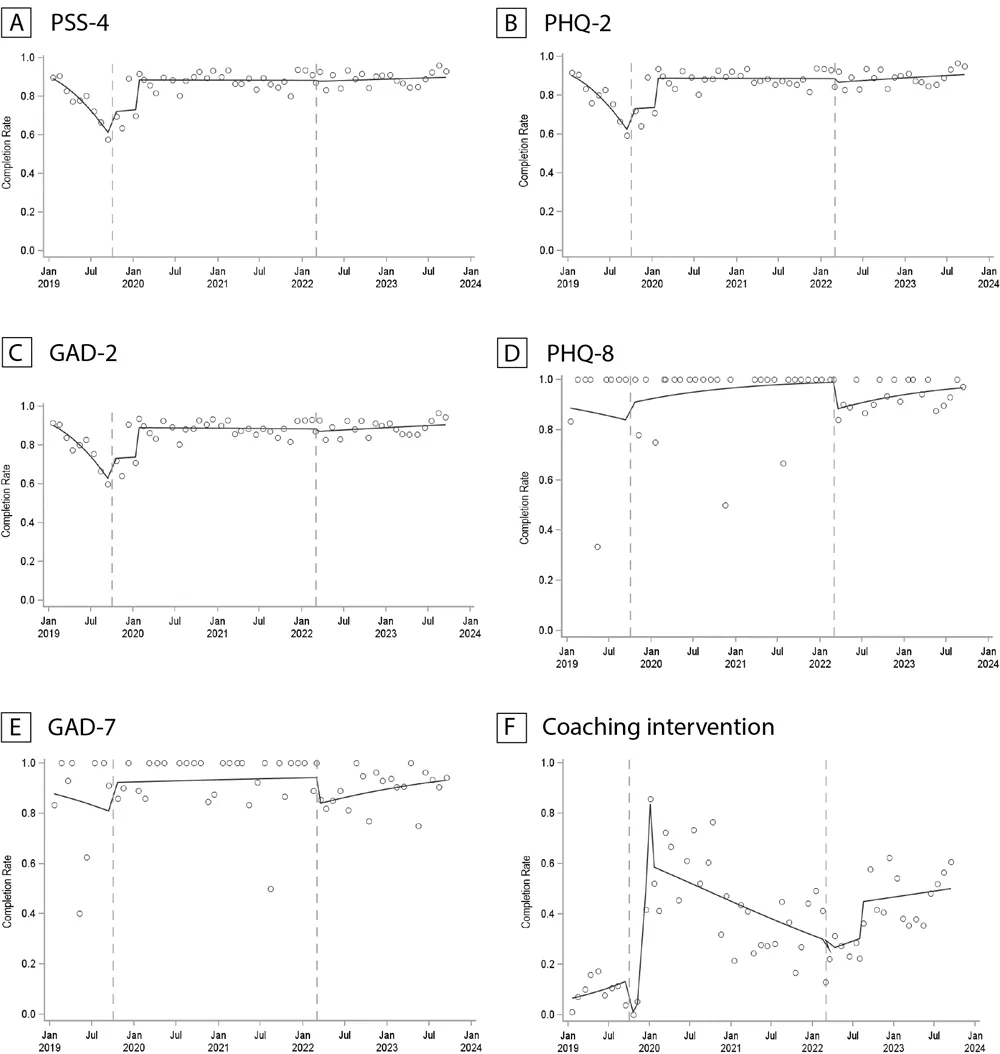
Interrupted time-series analysis (logistic regression) of behavioral health assessments and coaching intervention, Colorado Heart Healthy Solutions, 2018–2023. Vertical dashed lines denote interruption events with the first line representing the program adaptations in October 2019 and the second line representing those in March 2022. Circles represent observed completion rates aggregated to 30-day intervals. The solid lines denote predicted trend based on the unadjusted regression model. Abbreviations: GAD, Generalized Anxiety Disorder questionnaire; PHQ, Patient Health Questionnaire; PSS, Perceived Stress Scale.

Throughout the 5-year program, 51.9% of participants with PHQ-8 or GAD-7 scores of 10 or more had or were connected to care (Table 2). Although a negative trend was found over time, no indication of any relationship between connection to care and the 2 program adaptations was evidenced.

Use of the coaching intervention was low during the initial 10 months of implementation (8.2%). The addition of the simplified “Road Maps for Change” led to a 9-fold increase in the odds of completion compared with the initial implementation period (OR = 8.99; 95% CI, 4.51–17.93; *P* < .001) (Table 2 and Figure 2F). The estimated probability of completion of the intervention was 16.3% when the year 1 program adaptations were introduced and increased to 58.9% at the end of the 3-month adoption period (Figure 2F). However, use slowly decreased during years 2 and 3. With the addition of stress handouts in year 4, estimated probability of completion increased from 29.5% to 44.7% at the end of the 5-month adoption period (OR = 1.94; 95% CI, 1.42–2.63; *P* < .001).

### Maintenance

The estimated probabilities of completion of the PSS-4, PHQ-2, and GAD-2 assessments increased to 88.4%, 88.6%, and 88.8%, respectively, following the adoption period of the program adaptations in year 1 (Figure 2A–C). These estimates were sustained through 2023 during the rest of the project period, suggesting that CHWs were able to routinely integrate these assessments into their visits. Use of the intervention remained above 44.7% after the end of the adoption period of the year 4 program adaptations. Although not significant, the slope after the year 4 program adaptations may have indicated continued improvement (OR = 1.02; 95% CI, 0.99–1.04; *P* = .25), as completion rates in the final year were 42.8%.

## Implications for Public Health

This program is among the first to integrate multiple behavioral health components into a CHW-led lifestyle change program designed to improve CVD risk factors in rural communities. Assessing the feasibility of CHWs delivering the behavioral health components provides insights into how to optimize implementation.

### Reach of behavioral health components

Since its inception, CHHS has focused on delivering services to underserved populations by recruiting people through various community outreach and organizational referrals. However, we found differences in engagement between groups. The lower engagement rates among Hispanic participants in CHHS mirror national patterns: Hispanic adults are more likely to omit mental health items, underreport psychologic distress, and demonstrate lower completion rates for depression and anxiety screeners (37). Cultural norms surrounding emotional disclosure, stigma related to mental health labeling, and concerns about privacy likely contribute to these differences (38). Despite these challenges, behavioral health participation rates among Hispanic (85.7%) and Spanish‑speaking (84.7%) participants exceeded typical national benchmarks (39). This finding suggests that CHWs, who share similar backgrounds and life experiences with participants, may help reduce long-standing barriers to mental health disclosure in rural communities.

### Implementation of behavioral health components

The success of CHWs’ delivery of the behavioral health assessments, connections to care, and interventions can be attributed to the ongoing training and program adaptations. Early assessment completion rates (78.6%–79.9%) were consistent with rates described in previous studies but declined during the first year (40). This trend is consistent with challenges, such as time constraints, workflow disruption, and limited organizational support, commonly encountered during the early stages of implementing evidence-based practices (41). Additional barriers contributing to the decline may also have included stigma and limited experience in delivering behavioral health interventions among CHWs. The significant reversal in the decline in assessment completions after program adaptations were implemented during the training in year 1 may have been facilitated by more flexibility around the delivery of the assessments, allowing CHWs to choose between self-administered paper forms or verbally administered questions to help address barriers and adapt the behavioral health component to best serve their population.

Although the change in thresholds of PHQ-8/GAD-7 administration tripled eligibility and significantly increased the denominator of participants eligible for connection to care, we felt it was preferable to screen more people than have fewer false-positive screening results, as the change also helped identify people with milder symptoms, which aligned with the overall focus on prevention of chronic conditions. A review of preliminary data from our group-based lifestyle change program that administers the full PHQ-8 to all participants found that 31.3% of participants who screened positive on the PHQ-8 would have been missed if the score threshold for completing the PHQ-8 was 3 or more. Manual scoring further contributed to missed assessments. Reinforcing use of the OSCAR system restored completion rates, underscoring the importance of decision-support tools in sustaining fidelity.

In 2016, the US Preventive Services Task Force recommended routine depression screening for adults to facilitate timely diagnosis and treatment (42). However, actual screening rates in primary care are inconsistent, with studies reporting rates ranging from 59% in a large integrated health system using electronic clinical decision support to 67% among uninsured primary care patients (43,44). With the overall screening rates above 80%, incorporating mental health screening into community-based programs may be an effective strategy to identify people who might otherwise be missed in traditional health care settings and facilitate more timely connection to appropriate behavioral health care.

In this program, 51.9% of participants screening positive on the PHQ-8, GAD-7, or both (score of ≥10) were connected to behavioral health services. Although CHWs play an important role in facilitating these connections, linkage to care is influenced by both personal beliefs and practical access barriers (45). Common attitudinal barriers include the perception that help is unnecessary, mistrust of mental health providers, and doubts about treatment effectiveness. Practical barriers such as cost, distance from services, and caregiver responsibilities also limit follow-up. Additionally, in our intervention, referral pathways varied across CHHS communities; some CHWs were able to connect participants to internal behavioral health resources, whereas others relied on external providers, which may have further affected connection rates. It was also beyond the scope of this study to systematically verify use of services after referral.

The goal of our intervention was to help participants better understand and address unhealthy behaviors by exploring their emotional triggers and their readiness for change. However, use of the first iteration of the behavior maps was low. The adaptations made to the coaching interventions focused on feedback from the CHWs regarding the limited amount of time they had to complete behavior maps with participants and behavioral health topics participants were most comfortable discussing. In addition to being asked to identify barriers to implementation, the CHWs were asked to provide input on common behaviors, triggers, and stories that would be beneficial to address in the road maps and stress management interventions. Both intervention adaptations significantly increased use of the interventions by the CHWs. Overall, the program benefited from being receptive to how changes in the program can affect workflow and how CHWs can be supported when making program adaptations.

### Maintenance of behavioral health components

The maintenance benchmarks described in this assessment provide clear standards for evaluating long-term integration and align with implementation science standards, such as the Universal Stages of Implementation Completion, which define sustainment as continued service delivery for at least 2 years post-competence (32). By years 4 and 5, CHWs consistently exceeded sustainment benchmarks, with assessment completion rates above 89% and intervention delivery reaching 42.8%. Although referral rates remained stable, the program demonstrated durable integration of behavioral health components across diverse rural settings. These findings align with implementation science standards emphasizing adaptation, feedback, and ongoing support as critical to long-term sustainability.

### Limitations

This evaluation of the delivery of behavioral health components has several limitations. First were data collection problems, including data entry inconsistencies and data system constraints. For example, the data system was not designed to differentiate among participants who were already connected with care, participants who were referred to care by the CHWs, and participants who declined a referral. In addition, data entry was performed by CHWs across different organizations, which may have led to inconsistencies with how data were captured. These inconsistencies may have underestimated the proportion of participants who received the intervention, as well as those connected to care, as referrals to behavioral health providers may have been captured as primary care referrals when behavioral health resources were limited. Future iterations will better capture the range of support CHWs provide to participants, such as identifying other types of behavioral health resources (eg, personal support, educational materials) and encouraging healthy behaviors such as physical activity.

Second, implementing the intervention involved an iterative process adapting behavioral activation — originally designed for clinical treatment of depression — for use in a community setting (46). This adaptation required ongoing, incremental adjustments beyond the 2 program adaptations, which may have influenced the outcomes observed in the ITS analysis.

Third, because CHWs tailored program delivery to align with their local infrastructure and community assets, implementation of behavioral health components varied. This variability will be examined in future evaluations. Finally, the lack of a control group limits the generalizability of the findings.

### Conclusion

This CHW-led program demonstrates the feasibility of integrating multiple behavioral health components into a cardiovascular health lifestyle change intervention in rural communities. Incorporating behavioral health assessments, connections to care, and coaching interventions can enable CHWs to address a broader range of CVD risk factors and health behaviors, align support with participant needs and preferences, and strengthen motivation for cardiovascular health improvement. Embedding mental health into routine health conversations may also help to shift social norms and reduce stigma in rural settings.

The iterative data-informed approach, guided by continuous CHW feedback, helped balance fidelity and real-world implementation demands. This process enabled CHWs to consistently conduct assessments, facilitate connections to care, and deliver interventions despite challenges such as stigma, workforce disruptions, and the COVID-19 pandemic. Future directions include evaluating the impact of these behavioral health components on CVD outcomes, which will provide evidence for the effectiveness of integrated approaches for improving both mental and physical health in rural populations.
